# Quantifying Responses of Dung Beetles to Fire Disturbance in Tropical Forests: The Importance of Trapping Method and Seasonality

**DOI:** 10.1371/journal.pone.0026208

**Published:** 2011-10-18

**Authors:** Rafael Barreto de Andrade, Jos Barlow, Julio Louzada, Fernando Zagury Vaz-de-Mello, Mateus Souza, Juliana M. Silveira, Mark A. Cochrane

**Affiliations:** 1 South Dakota State University, Brookings, South Dakota, United States of America; 2 Lancaster University, Lancaster, Lancashire, United Kingdom; 3 Universidade Federal de Lavras, Lavras, Minas Gerais, Brazil; 4 Universidade Federal de Mato Grosso, Cuiabá, Mato Grosso, Brazil; Université Paris 13, France

## Abstract

Understanding how biodiversity responds to environmental changes is essential to provide the evidence-base that underpins conservation initiatives. The present study provides a standardized comparison between unbaited flight intercept traps (FIT) and baited pitfall traps (BPT) for sampling dung beetles. We examine the effectiveness of the two to assess fire disturbance effects and how trap performance is affected by seasonality. The study was carried out in a transitional forest between Cerrado (Brazilian Savanna) and Amazon Forest. Dung beetles were collected during one wet and one dry sampling season. The two methods sampled different portions of the local beetle assemblage. Both FIT and BPT were sensitive to fire disturbance during the wet season, but only BPT detected community differences during the dry season. Both traps showed similar correlation with environmental factors. Our results indicate that seasonality had a stronger effect than trap type, with BPT more effective and robust under low population numbers, and FIT more sensitive to fine scale heterogeneity patterns. This study shows the strengths and weaknesses of two commonly used methodologies for sampling dung beetles in tropical forests, as well as highlighting the importance of seasonality in shaping the results obtained by both sampling strategies.

## Introduction

Understanding how biodiversity responds to environmental changes is essential to provide the evidence-base that underpins conservation initiatives [1]. However, the understanding of consequences of habitat disturbance or the relative conservation value of different land-uses is complicated by many factors, including differences in the responses of different taxa, biodiversity metrics chosen, shifting baselines, and context specific results [2]–[4]. In this paper, we focus on an additional problem, that of sampling methodology, which can often complicate assessments of human impacts on biodiversity [5]–[7]. The objective of this study is to provide a large scale and standardized comparison between two common methods for sampling dung beetles, unbaited flight intercept traps (FIT) and baited pitfall traps (BPT). We chose fire disturbance and seasonality as scenarios for testing the congruence of the two methodologies.

Among human impacts, forest fires are considered a major threat to tropical natural environments [8]–[10], affecting vegetation structure, local biodiversity and forest dynamics [11]–[15]. Every year, thousands of square kilometers in the Amazon are affected by forest fires [10], [16], aggravated by deforestation and climate change [8]–[10]. Studies on tree mortality and forest structure (see [11] for review), understory avian communities [12], fruit production and large vertebrates [13] and invertebrates [17] show the multiple consequences of fires in the humid Neotropics. Seasonality is known to strongly affect invertebrate communities in the tropics [18]. Changes in precipitation and temperature are key factors for population dynamics and abundance of invertebrates [19], [20]. Hence, seasonality can play a significant role in biodiversity parameters when assessing disturbance impacts on invertebrates [21], [22].

Invertebrates use an array of microhabitats and are a key element in a number of ecosystem processes [23], [24], responding rapidly to environmental changes [25]–[28]. Among them, dung beetles (Coleoptera: Scarabeinae) are considered to be cost-effective indicators of anthropogenic disturbance [29], [3], and several studies have described their responses to a continuum of types and severity of environmental changes [2], [3], [6], [30]. Vegetation structure and climatic seasonality, especially rainfall variations, are also known to strongly affect dung beetle community structure [31]–[35]. Although little is known on the effects of fire on neotropical dung beetles, studies in other tropical habitats show this type of disturbance is an important factor affecting abundance and community composition of coleoptera [22], [36], [37].

A variety of collecting methods have been employed for dung beetles, including pitfall and flight interception traps, as well as light traps and direct searching in leaf-litter and other substrates. Baited pitfall traps (BPT), a have been extensively used in dung-beetle surveys, taking advantage of their strong flight capability and the fact that they actively search for food by odor-plumes [38]. Although baited sampling methods are convenient, they are also susceptible to a number of factors: for example, different sizes and types of bait could sample different dung-beetle assemblages [39]–[42]; decaying insects can decrease trap efficiency for coprophagous species, even within the first 24 h [40]; wind and temperature may affect bait effectiveness and the potential sampled area around each trap; and species are neither equally sensitive to bait odors nor of the same dispersal capabilities. Flight intercept traps (FIT), also known as window traps, sample dung-beetles without the use of bait and therefore may avoid some of the problems associated with baited traps (although decaying insects can act as an attraction to necrophagous species). However, they are not without their own series of disadvantages. Besides requiring longer sampling times in the field, FITs are less likely to capture species with lower flight frequencies and distance traveled per flight [43], and the sampling effectiveness is susceptible to changes in dung beetle activity, which are likely to occur if disturbance changes forest microclimate. Furthermore, it is known that FITs demand longer sampling periods for surveying a representative assemblage of dung-beetles [32].

Previous studies using both FITs and BPTs indicate that the two methods should be considered complementary, as they usually sample distinct components of the local beetle assemblage [33], [44]. However, due to time and logistical constraints it is not always possible to employ more than one method. Understanding the strengths and weaknesses of each method is vital for methodological decisions in biodiversity surveys, especially as dung beetles are increasingly used as indicators of human impacts in tropical ecosystems [3], [6]. To our knowledge, so far there have not been any standardized method comparison studies in tropical environments, comprising disturbed habitat responses.

Here, we provide the first standardized and quantitative comparison between two commonly used sampling methodologies for dung beetles, unbaited flight intercept traps (FITs) and baited pitfall traps (BPTs). We also examined the effectiveness of the two methodologies to assess fire disturbance impacts, and how this is affected by seasonality. Although we do not compare material costs or workforce required, since BPTs undoubtedly require simpler materials, we provide a quantitative comparison of the sampling efficiency of these two traps. We address the following specific hypotheses:

1. The two methodologies (FITs and BPTs) sample complimentary parts of the dung beetle assemblage, and there would be a significant difference in the biodiversity metrics recorded by each method (species richness, community composition, and rank abundance of species).
2. Both methodologies are equally effective at detecting disturbance in tropical forests - which in our case was forest fires - and there should be no difference in the number of traps required to find a significant difference in community composition between burned and unburned forest.
3. Seasonality will have a strong influence on the ability of the sample data to differentiate between unburned and burned forests, but FITs and BPTs will be equally affected.
4. The environmental factors recorded around each of the trap locations will be better predictors of the sample data from FITs than the sample data from BPTs. We based this hypothesis on the expectation that FITs will sample beetles from a smaller (more local) area of forest than BPTs, since individuals are not initially attracted to the traps.

## Methods

### Study site

The study was carried out in two, approximately 20 km^2^, forest fragments in the municipality of Querência (S 12°40′ W 52°21′), Mato Grosso state, Brazil (arthropod collection permission from IBAMA - Instituto Brasileiro do Meio Ambiente e dos Recursos Naturais Renovaìveis - #1029-1). The region is in the transitional region between Amazon forest and Cerrado (Brazilian savanna), and the vegetation in the undisturbed forests in the study region was characterized by closed canopy-forest with trees reaching 18–20 m [45]. Climate is characterized by a pronounced dry season from May to September with a mean annual rainfall of around 1500 mm [46]. Although some parts of one fragment had been affected by severe recurrent forest fires, for comparative purposes, we restricted sampling to areas affected by a single wildfire that occurred during the 2007 dry season, one year prior to the first sampling. Although we don't know the exact area affected by fire in the fragment, we used an adjacent area of unburned forest that the local farmer managed to protect from the fire by creating firebreaks as a control.

### Sampling methods

Dung beetles were collected in both the dry season (June 2008) and the wet season (February 2009). Eight 500 m transects, at least 500 m apart, were cut into the forest and four trapping points were placed at 50 m, 200 m, 350 m and 500 m along each transect. The 150 m between each trap avoids trap competition and guarantees independence of sampling points (Larsen & Forsyth 2005). Transects were marked with 50 m measuring tapes and the location of points where traps were placed were recorded with a GPS.

Each FIT, modified from [47], consisted of a 1 m high by 2 m wide nylon mesh screen with a plastic rain-cover suspended over it. The screen was placed vertically above plastic trays so that insects flying into it would fall into the saturated salted water and detergent contained in the trays. The lower end of the screen was no higher than 5 cm above the trays. Insects that fell into the FITs were collected after seven days and the trap dismounted. BPTs, modified from [48], were baited with human feces. Each pitfall consisted of a 1 liter, 15 cm wide, 9.5 cm deep plastic recipient buried at ground level and half-filled with saturated salted water and detergent. A small bag made of cotton gauze containing 20–30 g of human feces was suspended above the pitfall with a wooden stick. The lid of the plastic container was placed 10 cm above ground level with three wooden sticks, helping protect both the bait and the pitfall from rain. All insects captured in BPTs were collected after two days, a sampling period successfully used in recent biodiversity assessment studies ([49]–[51], among others). The saturated salted water solution minimizes the decay of trapped insects, though it does not completely prevent it. We chose to use sampling periods of seven days for FITs since it is known that this type of trap requires a longer time to capture a representative sample [26] and this period was used by previous studies [43]. At each trapping point, we used a FIT followed by a BPT. We did not use both trap types at the same time to avoid interaction and we used the FIT first, since this method does not actively attract coprophagous beetles and would have no significant impact on the subsequent BPT efficiency. All dung beetles were pinned and dried. Beetle processing was carried out at the Universidade Federal de Lavras, with identifications confirmed at Universidade Federal de Mato Grosso, and voucher specimens were deposited in both institutions.

### Environmental data sampling

For each transect, we censused a 0.50 ha forest plot (10×500 m). All trees with DBH above 10 cm and all lianas with DBH above 5 cm were recorded and measured for basal area estimations. Numbers of dead and live trees and stems were recorded in two subplots (5×5 m each) for each sampling point. In order to record the differences in canopy openness, we took 120° hemispherical photographs above each trap. Litter volume was calculated as a mean estimated volume of four 50 cm×50 cm samples of litter in a 40 cm×40 cm cylinder, at each sampling point. All environmental data was collected during the dry season, except litter volume, which was collected in both seasons.

### Data analysis

Species accumulation curves, as preliminary abundance and richness results, were made using Mao Tao estimator with 500 randomizations (EstimateS v. 7.52 software, [52]). For community composition analysis, we used similarity matrices generated using the Bray-Curtis similarity index. All data was standardized by sample size and log (x+1) transformed. For hypothesis 1, we used nonmetric multidimensional scaling (MDS) plots and Anosim tests to compare community composition between the two methods (Primer software v. 6.0, [53]). Mantel tests were used for testing composition and structure correlation and Spearman to test for abundance and richness correlation between methods and between seasons. We also listed the species exclusively captured by each trap type in both seasons.

To test hypothesis 2, we also used MDS plots and Anosim tests to compare community composition between burned and unburned forests, using both methods in the two seasons. Species abundance ranks, plotted according to [54], were used to illustrate dominance patterns. To compare how each trapping technique was able to distinguish between burned and unburned forest, we carried out Anosim tests with increasing trap numbers, since larger samples are expected to provide higher statistical power in this test. We used the mean *p* value out of five randomizations of trap numbers, except for the test using all 16 available traps that allowed no randomization. To test hypothesis 3, we compared the general results between the two sampling seasons and performed the Mantel and Spearman tests comparing composition, structure, abundance and richness. For hypothesis 4, we used Bioenv test [55] in Primer software v. 6.0 (Primer-E Ltd. 2006) to correlate community structure with environmental data. This procedure finds the best matching coefficient between the similarity matrices generated from the habitat variables sampled and that generated from the dung beetle data. All environmental data were standardized and tested with the Bray-Curtis similarity matrices. Canopy openness within photographs was analyzed using Gap Light Analyzer software (Simon Fraser University, Institute of Ecosystem Studies 1999). Because most analytical techniques cannot accept rows with zero individuals, it was necessary to exclude a small number of empty traps from the data analysis. As this affected both FITs and BPT traps in almost the same way (see below), we do not believe that these missing data bias our comparison between techniques.

## Results

**Figure 1:**
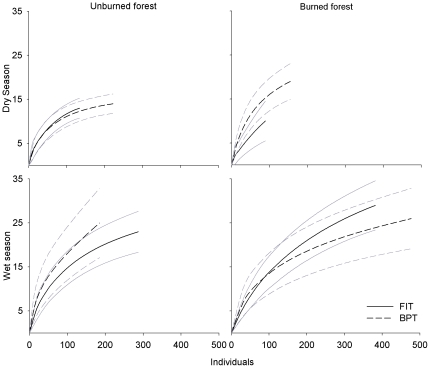
Randomized individual-based species accumulation curves. Samples from unburned and burned forest, using FIT and BPT methods, in two seasons. Grey lines are 95% confidence intervals.

A total of 1,931 beetles of 51 species were collected in both trapping methods in the two sampling seasons, with 892 in FITs and 1,039 individuals in BPTs (Table 1). The lowest total number of individuals (89) and species (9) were captured in FITs in burned forest during the dry season. The highest diversity was observed in FITs in burned forest during the wet season, with 29 species from 382 individuals. The highest number of individuals was observed in BPTs in burned forests during the wet season, with 477 individuals. During the dry season, a total of six FITs and five BPTs captured no individuals in burned forest. During the wet season, one BPT in each treatment (burned or unburned forest) captured no individuals. Species accumulation curves by individual (Fig. 1) show only FIT samples in unburned forest during dry season getting close to an asymptote.

**Table 1:** Individuals collected by the two trap types in dry and wet sampling seasons, in unburned and burned forests.

	Unburned forest		Burned forest	
Season	FIT	BPT	FIT	BPT
Dry	8.37 (2.0)	13.87 (2.4)	8.09 (2.3)	12.92 (4.8)
Wet	17.93 (3.7)	12.33 (2.2)	22.47 (11.7)	31.80 (4.6)

Mean per trap (standard error).

**Figure 2:**
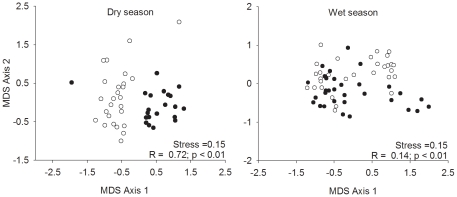
MDS ordination plots for FIT (black dots) and BPTs (white dots) community composition. Plots based on Bray-Curtis similarity on standardized and log_(X+1)_ transformed data at trap level. Test results based on Anosim test.

**Figure 3:**
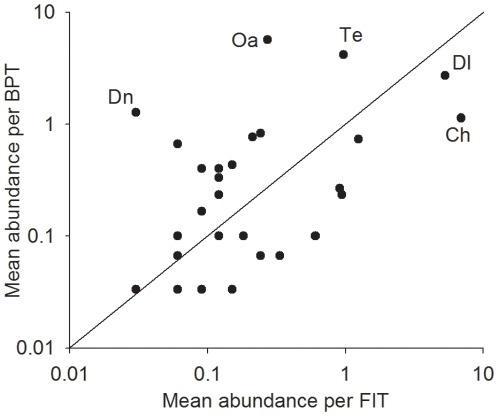
Mean abundance per trap for each species using the two methods. Dn = *Dichotomius nisus*; Oa = *Ontherus apendiculatus*; Te = *Trichillum externepunctatum*; Dl = *Dichotomius aff. lucasi*; Ch = *Canthidium aff. humerale*.

Our first hypothesis was generally given strong support by the data. In each season, both FIT and BPT captured species that were exclusive to that trap type (Table 2). Two out 9 exclusive wet season BPT species were captured by FITs in the dry season, and 8 out of 11 exclusive dry season BPT species were captured by FITs in the wet season. Only one exclusive dry season FIT species (*Eurysternus foedus*) was captured by BPT in the wet season. Out of composition, structure, abundance and richness parameters, only composition and structure, during the wet season, showed significant correlation between the two trapping methods (Table 3). Comparisons of community structure and composition using MDS and Anosim tests (Fig. 2) indicate that FIT and BPT sample different portions of the local dung beetle assemblage in unburned forest during both seasons. The five most abundant species were differently represented by the two methods (Fig. 3).

**Table 2:** Species exclusively captured by each trap in wet and dry seasons (*Species captured by the other trap type in the other season).

Wet season		Dry season	
FIT	BPT	FIT	BPT
*Canthidium sp. 1*	Canthon aff. pilluliformis*	*Anomiopus aff. pereirai*	Ateuchus sp.1*
*Canthidium sp. 2*	*Canthon sp.*	*Anomiopus batesi*	*Canthidium aff. ardens*
*Canthon aff. sericatus*	*Coprophanaeus dardanus*	*Anomiopus sp.1 gr. foveicollis*	Deutochilum orbiculare*
*Eutrichillum sp.*	*Dichotomius sp.gr. fissus*	*Canthidium aff. lentum*	Dichotomius aff. imitator*
*Uroxys sp.2*	*Eurysternus harlequin*	Eurysternus foedus*	Dichotomius melzeri*
	*Eurysternus howdeni*		Ontherus appendiculatus*
	Onthophagus aff. bidentatus*		Onthophagus aff. hirculus*
	*Onthophagus melzeri*		Oxysternon macleayi*
	*Pseudocanthon aff. xanthurus*		*Oxysternon silenus aeneum*
			*Pseudocanthon aff. xanthurus*
			Trichillum externepunctatum*

**Table 3:** Correlation test results for four different response methods, comparing seasonality and sampling methods (FITs and BPTs).

	Composition	Structure	Abundance	Richness
Season				
Dry FIT vs wet FIT	−0.20	−0.20	0.17	0.08
Dry BPT vs wet BPT	0.07	0.08	−0.18	−0.07
Method				
Dry FIT vs dry BPT	−0.09	−0.20	0.08	0.21
Wet FIT vs wet BPT	0.32*	0.38*	−0.14	−0.09

Mantel test was used for composition and structure and Spearman correlation test was used for abundance and richness.

*p<0.05.

**Figure 4:**
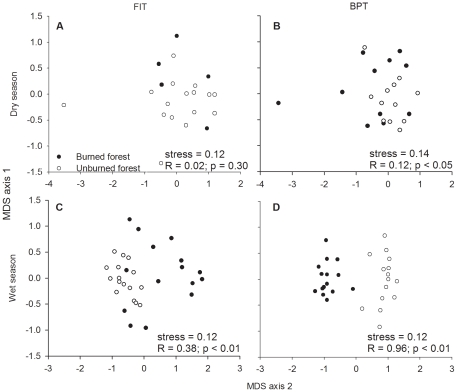
MDS ordination plots for burned/unburned forest communities for each sampling method in two seasons. Plots based on Bray-Curtis similarity on standardized and log_(X+1)_ transformed data at trap level. Test results based on Anosim test.

**Figure 5:**
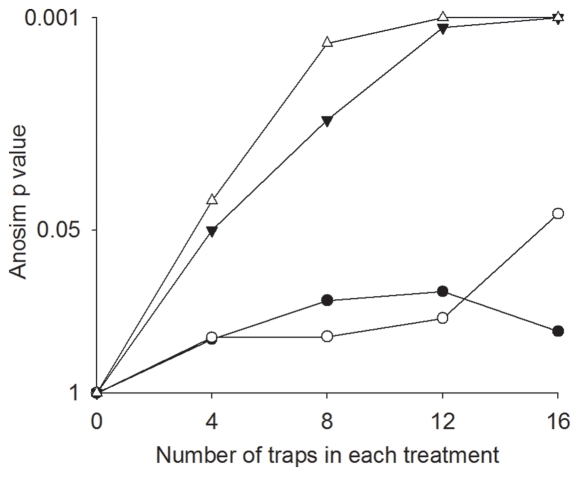
Mean Anosim level of significance (and SE) in different sample sizes using FITs and BPTs. Black dots are FIT in dry season, white dots are BPT in dry season, black triangles are FIT in wet season and white triangles are BPT in wet season.

The analysis also lends limited support for our second hypothesis that both methods would be equally effective at detecting changes in dung beetle community structure following forest fires. During the wet season, both methods were sensitive to fire disturbance, although the community composition plots sampled by FITs was more dispersed in burned forest (Figs. 4c and 4d). The separation between burned and unburned forest samples was much less apparent in both methods in the dry season (Figs. 4a and 4b), although only FIT samples in the dry season were not significantly different according to Anosim tests. In FIT samples, in dry season plots, seven points were clumped together after data standardization, since all seven samples collected only *Anomiopus sp.1 pr. foveicollis* individuals. Anosim tests using an increasing number of randomized traps (Fig. 5) shows that, during wet season, the BPT methodology is sensitive to fire disturbance with fewer sampling points than the FIT methodology. In the dry season, significance was only achieved with 16 sampling points in each treatment with BPTs. Species abundance evenness was similar between the two methods only during the wet season. During the dry season, FIT samples presented steeper slopes and dominance of fewer species, even though the distributions were not significantly different from BPT samples.

The results support our third hypothesis, that seasonality would strongly affect the ability of the sample data to detect fire disturbance, although BPTs were still reasonably effective in the dry season. We don't believe the eight-month interval between the dry and wet season samples allowed any significant recovery from the fire disturbance when compared to seasonality differences. Insect community recovery from fire disturbance spans several years in tropical forests [56].

Bioenv results (Table 4), correlating environmental factors recorded around each sampling location with the sample data from FITs and BPT (hypothesis 4), did not supported our hypothesis, revealing similar relationships between the environmental factors and samples in both types of trap. The percentage of dead stems and litter volume were the most correlated factors, followed by canopy openness and percentage of dead trees. Even though the correlation values were not particularly high and did not reveal a pronounced difference between the two methods, these community-environment relationships were strongly affected by seasonality, with much stronger relationships in the wet season (Table 4).

**Table 4:** Bioenv results for the two sampling methodologies in wet and dry seasons.

Method/Season	Best variable	r_s_	Second best variable	r_s_	Best combination of variables	r_s_
FIT/dry	DS	0.274	DT	0.057	DS	0.274
FIT/wet	LV	0.587	CO	0.562	LV, DT, DS	0.590
BPT/dry	LV	0.206	CO	0.143	LV, DT, DS	0.214
BPT/wet	DS	0.461	CO	0.456	DT, DS	0.567

DS = % Dead stems, DT = % Dead trees, LV = Litter volume, CO = % Canopy openness.

## Discussion

Our results indicate a series of differences between FITs and BPTs, many of which matched our hypotheses and expectations based on previous research. However, and importantly, we also show that, in this transitional forest, seasonality can have an effect as strong as trap type. We discuss these results, first comparing the results of the samples captured by each method, addressing peculiarities of species associated with each trap type. Then we discuss the efficiency of each method in assessing disturbance impacts and how seasonality affects the trapping results. Finally we examine the validity of our hypothesis that FITs are better at detecting community-environment relationships than BPTs.

Supporting our first hypothesis, the two methods sampled complimentary sets of the local beetle assemblage. Previous studies employing both methodologies also found complimentary arrays of species in each trap type [32], [43], [44], [57], [58]. Species accumulation curves suggest that a greater sampling effort could have captured a more representative portion of the local dung beetle assemblage and, perhaps, decreased differences between trap types. The significant differences in community composition captured with each trap (Fig. 2) indicate that the choice of methodology can bias the species proportions in the sample. This effect can be clearly observed, in our case, in species such as *Ontherus apendiculatus*, *Trichillum externepunctatum*, *Dichotomius aff. lucasi* and *Canthidium aff. humerale*, since they were commonly found in BPT samples and rare in FIT samples.

Species peculiarities may cause differences in the susceptibility to being captured by each trap type. Dietary preference is the most obvious factor, since, unlike FITs, BPTs attract coprophagous species. These include *Canthidium aff. ardens* [59], [60], *Dichotomius aff. lucasi* [61], *Dichotomius melzeri* [Vaz-de-Mello *pers. obs.*] and *Oxysternon silenus aeneum* [62, Vaz–de–Mello *pers. obs.*]. Despite the use of human dung, two species exclusively captured by BPTs, *Eurysternus harlequin* and *E. howdeni*, are commonly associated with large mammal dung (e.g. tapirs) [Vaz-de-Mello *pers. obs.*]. On the other hand, species known to have different feeding habits were also exclusively captured by BPTs, such as *Coprophanaeus dardanus*, a species that belongs to a mainly necrophagous group [63], *Dichotomius sp. gr. fissus*, a species likely to be frugivorous ([64], Vaz-de-Mello *pers. obs.*), and *Pseudocanthon aff. xanthurus*, which seems to be of generalist feeding habits (Vaz-de-Mello *pers. obs.*).

The feeding habits of the species captured exclusively by FITs are very poorly known, likely because these are rarely captured in baited traps that are more often used in ecological studies. These species include *Anomiopus aff. pereirai*, *Anomiopus batesi*, *Anomiopus sp. gr. foveicollis* ([65], Vaz-de-Mello *pers. obs.*). These results highlight the importance of this type of trap when conducting biodiversity surveys. The two species exclusive to FIT (*Canthidium aff. lentum* [Vaz-de-Mello *pers. obs.*] and *Uroxys* sp.) are known to be coprophagous. One of these belongs to a group usually associated to sloths [66], [67], and it may be that these species are not attracted by human dung. Additionally, the genus *Eutrichillum* is assumed to be strictly necrophagous, and could have been attracted by decaying insects in the trap [68]. If this was the case, and carrion smell influenced the beetles assemblage captured in FITs, then our conclusions on the complementarity of the two methods need to be considered accordingly. However, the genus *Eutrichillum* accounted for a relatively small proportion of the total number of captures (six out of 669 individuals in the wet season), and we consider it unlikely that attraction to carrion had a significant influence on results. However, daily collection of insects trapped in FITs can eliminate this problem, and further studies are needed to quantify the effects of decaying insects in these traps.

Both FITs and BPTs were effective at detecting disturbance in tropical forests during the wet season, but BPT appeared more effective as they required a smaller number of traps to detect a significant difference between communities from unburned and burned forests. The much larger area sampled by each BPT [33] may contribute to the efficiency of this trap in detecting large-scale patterns such as fire disturbance. BPT were also more effective during the dry season, when FIT data did not show a significant change in dung beetle community structure. These results highlight the importance of considering seasonality when evaluating the impact of disturbance on biodiversity, and support previous studies that show dung beetles are particularly sensitive to rainfall [31] and, the more pronounced seasonality is, the stronger the community responses to disturbance [69], [70]. These results also highlight some specific shortcomings of the FIT methodology, which are less effective during the dry season. There are a number of plausible explanations for this. During very dry conditions, active populations may decline to small “population pockets” concentrated in humid microhabitats [27], making it unlikely that unbaited trapping methods will detect them. Also, patrolling flight activity, in the absence of a direct odor source, may also decline during dry seasons. Another possibility is that the assemblage of species captured by FITs during dry seasons may be less susceptible to fire disturbance. Finally, our conclusions regarding field time efficiency are limited by the fact that we compared two-day samples from BPT with seven-day samples from FIT. Further studies comparing daily samples obtained by each trapping method (e.g. daily species accumulation curves) are necessary to provide more details on effectiveness and sampling effort required for the two methods.

Our results provide limited support for hypothesis 4, that FIT samples would be a better predictor of environmental factors. Despite the correlation values being slightly higher in both FIT samples, overall correlation was not particularly high. However, in MDS plots, BPT samples appear more clustered than FIT ones, showing community composition sampled by FITs is more variable than that sampled by BPTs. Similar results have been found by [44], and these patterns could indicate that FITs are more sensitive to fine scale patterns, such as community heterogeneity in a small area. While a flight intercept trap captures only the beetles that fly through the exact point of the trap, BPTs are likely to attract most individuals within an approximately 25 m radius [5], and may attract larger species far beyond that radius. Again, the strong effect of the dry season visibly decreased correlations with environmental variables in both methodologies.

### Conclusion

We reveal strengths and weaknesses of pitfall and flight intercept traps for conducting standardized dung beetle surveys and evaluating the impact of disturbance on tropical forests. BPTs provided a more representative sample of individuals under low population conditions (dry season) and required less sampling points to detect differences in dung-beetle community due to fire disturbance. Allied to logistical advantages, such as low-cost materials and quick sampling time, this makes BPTs a more cost-effective and robust methodology (c.f. [3]). The initially passive nature of FITs makes them more adequate for detecting fine scale patterns, and may be suitable when habitat and community heterogeneity are key factors. However, this methodology is less effective at detecting change resulting from habitat disturbance when beetle densities are low, and requires more complex materials and longer sampling periods. Our results also highlight the importance of seasonality in shaping the results obtained by both sampling strategies. For evaluating the impacts of forest degradation, the pronounced seasonality of this transitional region between Amazon and Cerrado vegetation appeared to be as important a factor as the trapping method. Different factors, such as methodology and average rainfall of the sampling season, must be considered when sampling dung beetles in a tropical region. Although there are some additional factors that can have a significant influence on the cost-effectiveness of each method (such as material price and availability, workforce required, etc cf. [3]), our study highlights some important features of each of these two trapping techniques, providing information regarding seasonality and sample effort that can be very helpful for study design. Further studies testing different number of sampling days for each trap type and controlling for the problem with decaying insects can provide more useful information concerning dung beetle sampling protocols.
